# ‘Laser chemistry’ synthesis, physicochemical properties, and chemical processing of nanostructured carbon foams

**DOI:** 10.1186/1556-276X-8-233

**Published:** 2013-05-16

**Authors:** Andrés Seral-Ascaso, Rosa Garriga, María Luisa Sanjuán, Joselito M Razal, Ruth Lahoz, Mariano Laguna, Germán F de la Fuente, Edgar Muñoz

**Affiliations:** 1 Instituto de Carboquímica ICB-CSIC, Miguel Luesma Castán 4, 50018 Zaragoza, Spain; 2 Departamento de Química Física, Universidad de Zaragoza, C/Pedro Cerbuna s/n, 50009 Zaragoza, Spain; 3 Instituto de Ciencia de Materiales de Aragón, Universidad de Zaragoza-CSIC, 50009 Zaragoza, Spain; 4 ARC Centre of Excellence for Electromaterials Science and Intelligent Polymer Research Institute, AIIM Facility, Innovation Campus, University of Wollongong, Wollongong, NSW 2522 Australia; 5 Instituto de Síntesis Química y Catálisis Homogénea, Universidad de Zaragoza-CSIC, Plaza San Francisco s/n, 50009 Zaragoza, Spain

**Keywords:** Carbon nanostructures, Laser ablation, Metal-carbon hybrids, Laser chemistry, Metal nanoparticles, Fiber spinning

## Abstract

Laser ablation of selected coordination complexes can lead to the production of metal-carbon hybrid materials, whose composition and structure can be tailored by suitably choosing the chemical composition of the irradiated targets. This ‘laser chemistry’ approach, initially applied by our group to the synthesis of P-containing nanostructured carbon foams (NCFs) from triphenylphosphine-based Au and Cu compounds, is broadened in this study to the production of other metal-NCFs and P-free NCFs. Thus, our results show that P-free coordination compounds and commercial organic precursors can act as efficient carbon source for the growth of NCFs. Physicochemical characterization reveals that NCFs are low-density mesoporous materials with relatively low specific surface areas and thermally stable in air up to around 600°C. Moreover, NCFs disperse well in a variety of solvents and can be successfully chemically processed to enable their handling and provide NCF-containing biocomposite fibers by a wet-chemical spinning process. These promising results may open new and interesting avenues toward the use of NCFs for technological applications.

## Background

Laser technologies can be successfully utilized for the production of carbon-nanostructured materials exhibiting fascinating structural and physical properties such as carbon nanotubes [1], carbon nanohorns [2], carbon nanofoams [3], or shell-shaped carbon nanoparticles [4]. Our group discovered the production of metal-nanostructured foams (NCFs) by laser ablation of triphenylphosphine (PPh_3_)-containing organometallic targets [5]. We then demonstrated that organic ligands can act as efficient carbon sources for the laser ablation production of carbon nanomaterials. Metal-NCFs are three-component materials which consist of amorphous carbon aggregates, metal nanoparticles embedded in amorphous carbon matrices, and graphitic nanostructures. The metal-NCF composition, metal nanoparticle size, and dilution (i.e., metal and carbon content) within the carbon matrices can be tailored by conveniently choosing the metals (Au, Cu) and ligands of the ablated targets [6]. On the other hand, laser ablation of PPh_3_ resulted in the production of metal-free NCFs consisting of graphitic nanostructures and P-containing amorphous carbon aggregates [6]. We report how our versatile ‘laser chemistry’ approach can be extended to the synthesis of a variety of other metal-NCFs, as well as to metal-free, P-free NCFs, proving that the synthesis of NCFs is not restricted to PPh_3_-based targets and therefore enabling envisioning the synthesis of metal-carbon hybrids by chemical design. Additionally, physicochemical studies have been performed on metal-free NCFs to evaluate their potential applications. We also show that NCFs can be easily chemically processed in the form of stable NCF dispersions in different solvents and NCF biocomposite fibers, which offer promise for NCF incorporation into different matrices and technological applications.

## Methods

**Figure 1:**
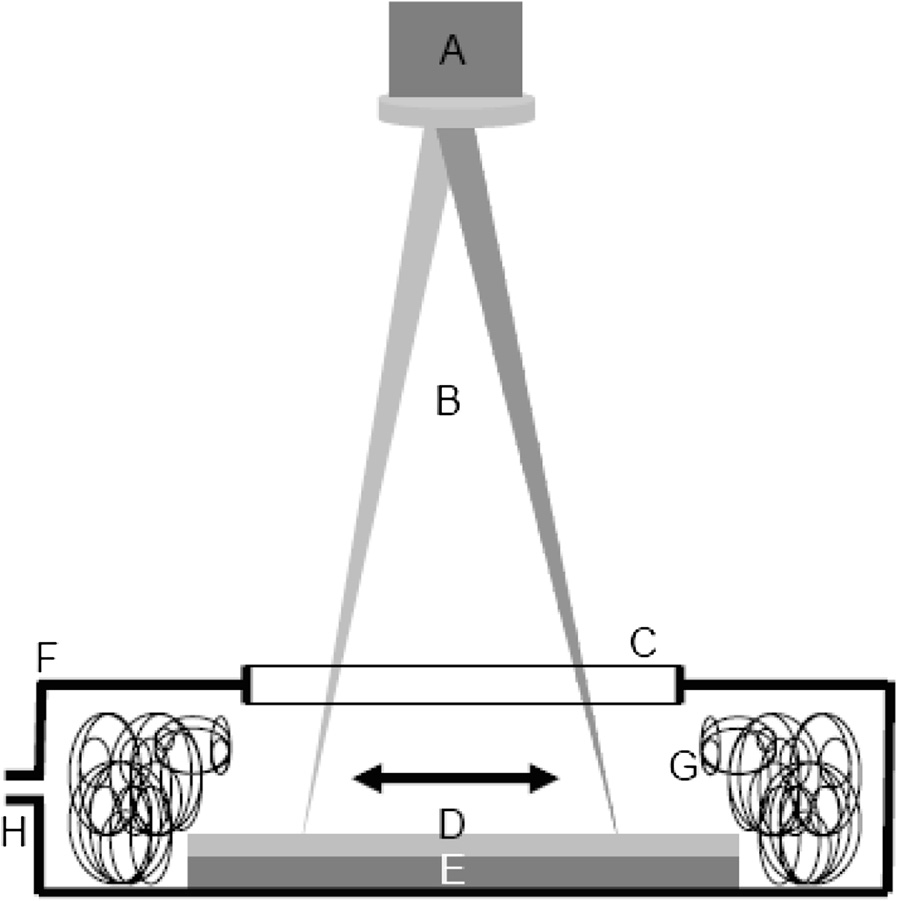
**Schematic diagram of the experimental setup used for the laser ablation production of NCFs.** A galvanometer mirror box (**A**) distributes the laser radiation (**B**) through a flat field focal lens and a silica window (**C**) onto layers of the employed organometallic compounds (**D**) deposited onto a ceramic tile substrate (**E**) placed inside a portable evaporation chamber (**F**). The synthesized soot is mainly collected on an entangled metal wire system (**G**). The produced vapors are evacuated through a nozzle (**H**).

The production of carbon foams has been carried out by Nd:YAG laser ablation of thick layers of coordination and organic compounds in air atmosphere using the setup described in Figure 1 and under the experimental conditions described elsewhere [5,6]. Different metal-NCFs have been produced by laser irradiation of dichlorobis(triphenylphosphine)nickel(II) [NiCl_2_(PPh_3_)_2_], dichlorobis(triphenylphosphine)cobalt(II) [CoCl_2_(PPh_3_)_2_], and [1,2-bis(diphenylphosphino)ethane]dichloroiron(II) [FeCl_2_(Dppe)]. P-free metal-NCFs were produced using bis(benzonitrile)dichloropalladium(II) [PdCl_2_(PhCN)_2_], dichloro(1,10-phenanthroline)palladium(II) [PdCl_2_(Phen)], and (2,2´-bipyridine)dichloropalladium(II) [PdCl_2_(Bipy)]. Naphthalene, phenanthrene, and 1,10-phenanthroline have been used as precursors for the synthesis of metal-free, P-free NCFs. All chemicals were purchased from Sigma-Aldrich (Schnelldorf, Germany and Saint-Quentin-Fallavier, France) and used as received.

The structure of the synthesized NCFs was imaged by scanning electron microscopy (SEM, Hitachi S-3400N (Hitachi, Ltd., Chiyoda-ku, Japan), including a Röntec XFlash detector (Röntec GmbH, Berlin, Germany) for energy dispersive X-ray spectroscopy (EDS) analyses), and transmission electron microscopy (TEM, JEOL JEM-3000F microscope, JEOL Ltd., Akishima-shi, Japan, equipped with an Oxford Instruments ISIS 300 X-ray microanalysis system and a Link Pentafet detector, Oxford Instruments, Abingdon, UK, for EDS analyses). NCF thermal stability in air was studied by thermogravimetric analysis (TGA, SETARAM Setsys Evolution, Hillsborough, NJ, USA; samples were analyzed in Pt pans at a heating rate of 10°C/min up to 850°C in an atmosphere of air flowing at 100 mL/min). Micro-Raman spectroscopy studies were carried out using a Dilor XY Raman spectrometer (*λ*_exc_ = 514.5 nm, HORIBA, Ltd., Kyoto, Japan). Elemental analyses of metal-free NCFs were performed using a Thermo Flash EA 1112 Series NC analyzer (Thermo Fisher Scientific, Waltham, MA, USA). The textural properties of NCFs were studied using nitrogen adsorption-desorption isotherms measured at 77 K (Micromeritics ASAP 2020, Norcross, GA, USA) and using the Brunauer-Emmett-Teller (BET) method between 0.05 and 0.3 P/P_0_ and t-Plot and Barret-Joyner-Halenda (BJH) method. Density values were measured using an AccuPyc II 1340 Micromeritics helium picnometer (Micromeritics, Norcross, GA, USA).

Fiber spinning of NCF biocomposites was performed by injecting 1:4 Au-NCF:sodium alginate (MW: 400K) aqueous dispersions (1 mg/mL Au-NCF prepared by bath sonication) into a coagulation bath (5% CaCl_2_ solution in 70% methanol) following the carbon nanotube biofiber spinning procedure reported by Razal et al. [7]. The electrical conductivity of the spun fibers was characterized by four-probe resistance measurements using a Keithley 2000 Multimeter (Keithley Instruments, Inc., Cleveland, OH, USA).

## Results and discussion

**Figure 2:**
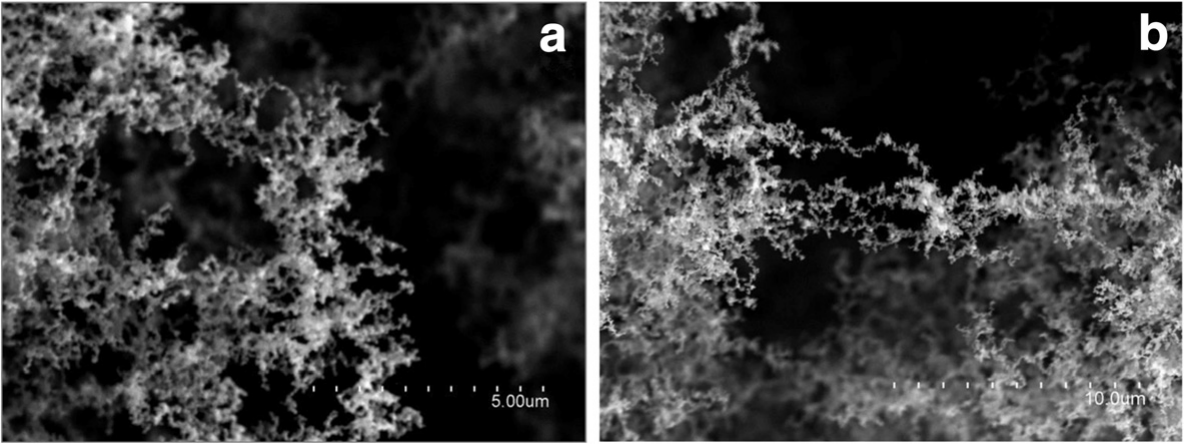
**SEM images showing the spongy microstructure of NCFs.** SEM micrographs of NCFs produced by laser ablation of [FeCl_2_(Dppe)] (**a**) and phenanthrene (**b**).

**Figure 3:**
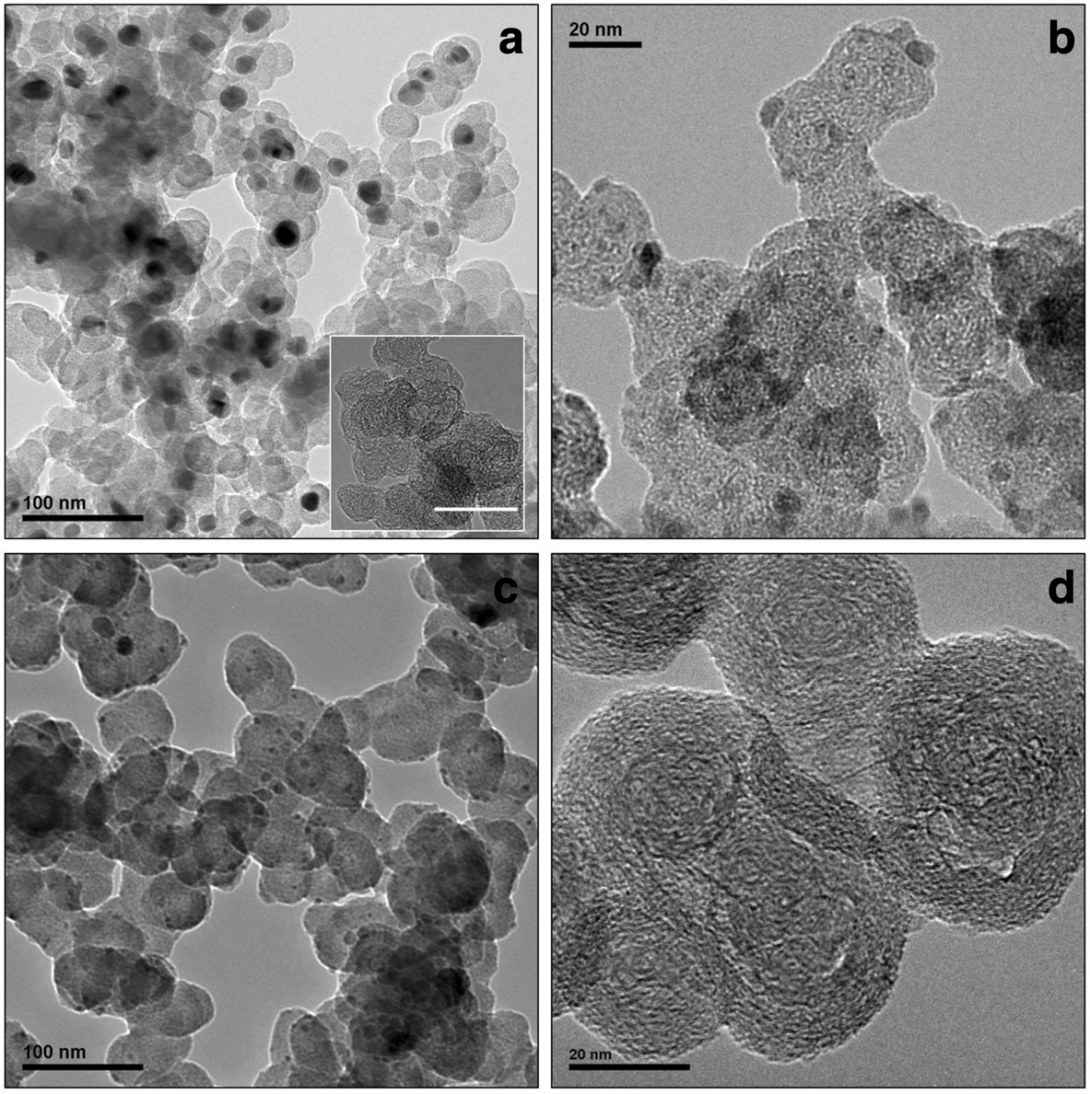
**TEM characterization of the different components of NCFs.** TEM images of NCFs produced using [PdCl_2_(PhCN)_2_] (**a**), [NiCl_2_(PPh_3_)_2_] (**b**), [CoCl_2_(PPh_3_)_2_] (**c**), and naphthalene (**d**) targets. Inset on (**a**) shows graphitic structures observed on [PdCl_2_(Phen)] foams (scalebar 50 nm).

SEM (Figure 2), TEM (Figure 3), and EDX characterization of the soot that resulted from the laser irradiation of different organometallic targets show that our laser ablation technique is not only restricted to the synthesis of Au/NCFs and Cu/NCFs [5,6], but it can also provide a new family of metal-NCF hybrids of any desired metal. These metal-NCFs exhibit a spongy-like microstructure (Figure 2a) as a result of nanoparticle assembly. These nanoparticles consist of amorphous carbon particles, graphitic nanostructures, and metal nanoparticle-containing amorphous carbon aggregates (Figure 3a,b,c). Moreover, metal-NCFs that result from the laser irradiation of [PdCl_2_(PhCN)_2_], [PdCl_2_(Phen)], and [PdCl_2_(Bipy)] also indicate that aromatic ligands different than PPh_3_ and without phosphor in their composition, such as benzonitrile, 1,10-phenanthroline, or 2,2´-bipyridine, can also efficiently act as carbon source for the laser production of carbon matrices (Figures 2 and 3).

**Figure 4:**
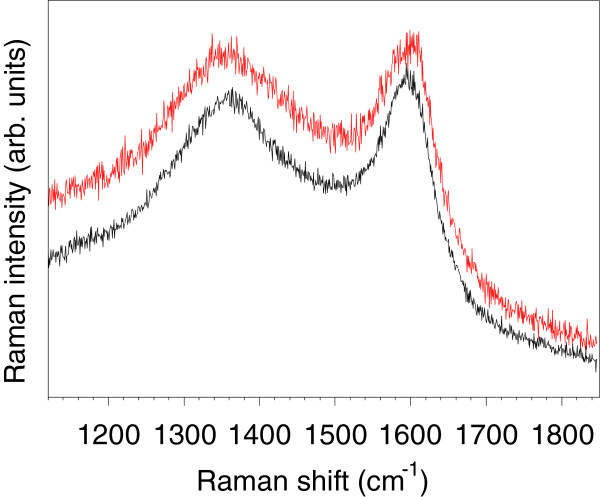
**Raman spectra show typical features of high degree carbon disorder in NCFs produced from naphthalene.** The high degree of carbon disorder in NCFs produced by laser ablation of naphthalene is also demonstrated by the presence of broad bands centered at approximately 1,360 cm^−1^ (D-band) and approximately 1,590 cm^−1^ (G-band) of equivalent intensities in Raman spectra.

Based on these findings, we then irradiated different aromatic compounds toward the synthesis of metal-free and P-free NCFs. Thus, laser ablation of naphthalene, phenanthrene, and 1,10-phenanthroline resulted in the formation of a NCF material which consisted of both amorphous carbon aggregates and graphitic nanodomains (Figures 2b and 3d). Elemental analysis data reveal high carbon contents (≥95%) for these metal-free NCFs. The extensive charging observed in NCFs without any conductive coating deposited on conducting carbon films for SEM characterization reveals the nonconducting nature of these materials. The Raman spectra of the metal-free NCFs show broad D- and G-bands of comparable intensities, a feature typical of short-range *sp*^2^-bonded carbons [6,8]. As an example, we show in Figure 4 the spectrum of NCFs produced by laser ablation of naphthalene. The much broader aspect of the D-band (as compared to the G-band) indicates that this material lacks long-range graphitic order. According to Ferrari's model of graphite amorphization path [8], this material would be in stage 2 of amorphization (denoted as *sp*^2^ a-C in [8]) in which only some *sp*^2^-bonded rings remain, thus confirming the predominance of amorphous carbon already observed by TEM.

TGA analyses show that metal-free NCFs are thermally stable in air up to temperatures of approximately 600°C. It is interesting to point out that the temperature of maximum decomposition rate of NCFs produced by laser ablation of PPh_3_ (which contains 8.2% P) is about 30°C higher than that of the naphthalene-produced NCFs, probably as a result of flame retardant role of P [9]. The study of the textural properties reveals that NCFs produced by laser ablation of PPh_3_ and naphthalene are mesoporous materials with BET surface areas between 33 and 63 m^2^/g and mesopore volumes of 0.046 to 0.168 cm^3^/g, respectively. The measured BET surface area values are lower than those of other carbon materials consisting of amorphous carbon aggregates such as carbon aerogels (typical values in the range 400 to 600 m^2^/g) [10,11] and carbon nanofoams (300 to 400 m^2^/g) produced by femtosecond pulsed laser ablation of HOPG [12]. Additionally, density values of 1.66 g/cm^3^ have been measured for naphthalene-produced NCFs by He picnometry. These values are similar to those of other carbon materials (Table 1) such as multi-walled carbon nanotubes, carbon xerogels, carbon black, graphitic cones, and ordered mesoporous carbon but significantly higher than those reported for carbon nanofoams produced by ultrafast lasers (0.02 to 0.002 g/cm^3^) [12].

**Table 1:** Measured densities of different carbon materials

**Carbon material**	**Density (g/cm**^**3**^**)**
NCF	1.66
Multi-walled carbon nanotubes^a^	1.98
Nanodiamond^b^	2.97
Graphitic cones^c^	1.96
Carbon aerogel	0.20 to 1.00 [10,11]
Carbon xerogel^d^	1.73
Carbon black^e^	1.91
Activated carbon^f^	2.05
Graphite^g^	2.27
Ordered mesoporous carbon^h^	1.63
Carbon nanofoam	0.020 to 0.002 [12]

^a^High-purity multi-walled carbon nanotubes produced by the CVD technique (10 to 15 nm in diameter, ≥10 microns in length; Nanothinx S.A.); ^b^Nanodiamonds, purified, grade G01 (PlasmaChem); ^c^Graphitic cones produced by hydrocarbon pyrolysis (n-TEC) [13]; ^d^Carbon xerogels prepared by polycondensation of resorcinol and formaldehyde in water by Pekala's sol-gel method [14]; ^e^Vulcan XC-72R carbon black (Delta Tecnic S.A.); ^f^Activated carbon (Morgui Clima S.L.); ^g^Graphite, particle size <50 μm (Merck); ^h^Ordered mesoporous carbon synthesized using a template-mediated process [15].

**Figure 5:**
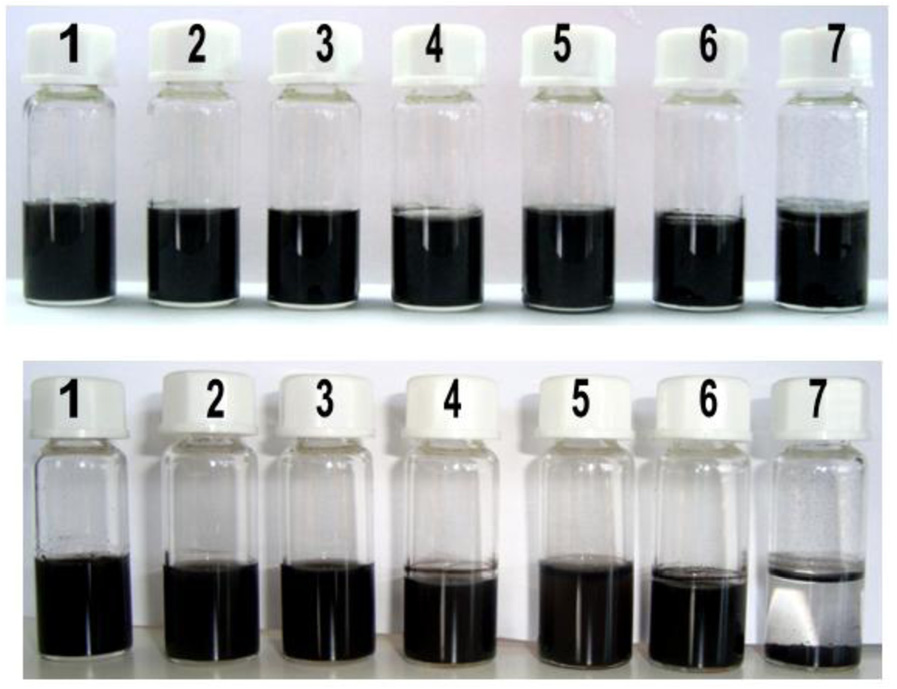
**NCFs easily disperse in various solvents.** Top image shows NCFs in different solvents 60 s after being dispersed by mild sonication. Bottom image shows the same dispersions after 48 h. Solvents: 1-water, 2-acetone, 3-ethanol, 4-diethyl ether, 5-toluene, 6-dichlorometane, 7-hexane.

**Figure 6:**
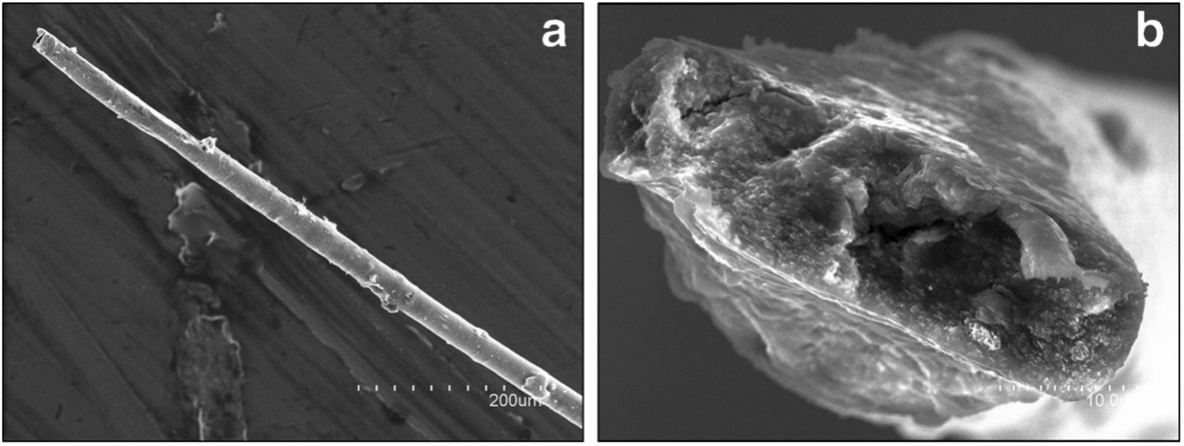
**SEM micrographs of Au-NCF/alginate composite biofibers.** SEM micrographs show a fiber overview (**a**) and the microstructure at the fiber cross-section (**b**).

NCFs are collected from laser ablation processes as intractable soots. In order to evaluate the potential chemical processing capabilities of our NCFs, these materials were dispersed in different solvents. Mild (bath) sonication resulted in NCF dispersions which are stable for over 48 h in all tested solvents but in hexane (Figure 5). This NCF remarkable dispersibility opens new opportunities toward the incorporation of these nanocarbons into functional materials and assemblies. Thus, Au-NCF/alginate biocomposite fibers, tens of centimeters in length and 30 to 50 micrometers in diameter (Figure 6), were spun by coagulation of sodium alginate assisted Au-NCF aqueous dispersions in a CaCl_2_ water/methanol solution, followed by RT drying in air of the resulting elastomeric gels. Four-probe resistance measurements revealed that these fibers were nonconducting. This fiber spinning method is an interesting strategy for easy NCF handling and for providing a confinement in the form of quasi 1D architectures to metal nanoparticles.

## Conclusions

The laser chemistry approach described in the present work is a versatile method for the synthesis of metal nanoparticles embedded in carbon matrices from molecular precursors. This laser chemistry is very appealing for applications requiring metal nanoparticles largely isolated from each other embedded in solid matrices. Moreover, it can be used for the synthesis of metal-free, P-free NCFs from commercial organic precursors, which would in turn facilitate upscaling their production. On the other hand, the chemical processing capabilities of NCFs ease their handling and may open attractive opportunities toward their incorporation into matrices and applications. Future challenges should deal with the design of production or processing strategies to increase the surface area and conductivity of these materials to enable their use as, for example, electrode materials, in catalysis, or as functional magnetic materials.

## Competing interests

The authors declare that they have no competing interests.

## Authors' contributions

ASA, RL, GFF, and EM carried out the laser ablation experiments. EM, GFF, ML, and ASA conceived the study. MLS performed the Raman characterization. ASA carried out the electron microscopy and physicochemical characterization, and completed the data analysis. RG was in charge of further physicochemical studies and assisted in data analysis. JMR and EM performed the fiber spinning experiments. RG and EM drafted the manuscript. All authors read and approved the final manuscript.
